# Transcriptome Analysis of Calcium- and Hormone-Related Gene Expressions during Different Stages of Peanut Pod Development

**DOI:** 10.3389/fpls.2017.01241

**Published:** 2017-07-14

**Authors:** Yan Li, Jingjing Meng, Sha Yang, Feng Guo, Jialei Zhang, Yun Geng, Li Cui, Shubo Wan, Xinguo Li

**Affiliations:** ^1^Biotechnology Research Center, Shandong Academy of Agricultural Sciences Jinan, China; ^2^Shandong Provincial Key Laboratory of Crop Genetic Improvement, Ecology and Physiology, Shandong Academy of Agricultural Sciences Jinan, China

**Keywords:** peanut, calcium, hormone, pod development, transcriptome

## Abstract

Peanut is one of the calciphilous plants. Calcium serves as a ubiquitous central hub in a large number of signaling pathways. In the field, free calcium ion (Ca^2+^)-deficient soil can result in unfilled pods. Four pod stages were analyzed to determine the relationship between Ca^2+^ excretion and pod development. Peanut shells showed Ca^2+^ excretion at all four stages; however, both the embryo of Stage 4 (S4) and the red skin of Stage 3 (S3) showed Ca^2+^ absorbance. These results showed that embryo and red skin of peanut need Ca^2+^ during development. In order to survey the relationship among calcium, hormone and seed development from gene perspective, we further analyzed the seed transcriptome at Stage 2 (S2), S3, and S4. About 70 million high quality clean reads were generated, which were assembled into 58,147 unigenes. By comparing these three stages, total 4,457 differentially expressed genes were identified. In these genes, 53 Ca^2+^ related genes, 40 auxin related genes, 15 gibberellin genes, 20 ethylene related genes, 2 abscisic acid related genes, and 7 cytokinin related genes were identified. Additionally, a part of them were validated by qRT-PCR. Most of their expressions changed during the pod development. Since some reports showed that Ca^2+^ signal transduction pathway is involved in hormone regulation pathway, these results implied that peanut seed development might be regulated by the collaboration of Ca^2+^ signal transduction pathway and hormone regulation pathway.

## Introduction

Peanut (*Arachis hypogaea* L.) is an important crop member of the legume family and a major source of plant oil, proteins, essential vitamins and minerals that can be used for human consumption, animal feed, bioenergy, and health products (Higgs, 2002; Li et al., 2010, 2011). Seed formation of peanut is a central stage of pod development. Some reports illustrated that seed development depends on the highly coordination between endogenous signal and environment stimuli (Sun, 2008). For instance, several plant hormones have long been known to play a significant role in peanut gynophore elongation and embryo differentiation, such as auxin (Jacobs, 1951; Moctezuma and Feldman, 1996), the ration of NAA and kinetin (Ziv and Zamski, 1975), ABA (Ziv and Kahana, 1988), ethylene (Shlamovitz et al., 1995), and so on. In addition, peanut needs more calcium relative to other plants. Free Ca^2+^ concentration in soil serious affects peanut fruiting and yield (Cox et al., 1982). During pod development, more than 90% calcium was directly absorbed from the soil by pod (Beringer and Taha, 1976). For plants, Ca^2+^ not only play roles as a nutrient element, but also as a second messenger in the regulation of diverse metabolic processes (Poovaih and Ready, 1993). As to the possible relationship between calcium signal transduction and plant hormones, Yang et al. (2015) reported that the expressions of most calmodulin-binding transcription activators of *M. Truncatula* (MtCAMTAs) were responsive to the four hormones, including IAA, salicylic acid (SA), jasmonic acid (JA), and ABA, which play critical roles in the regulation of nodule organogenesis in legumes. Additionally, it seems that the ripening of strawberry fruit are co-regulated by phytohormone and calcium signal transduction (Chen et al., 2016). Recent years, although there is a comprehensive understanding of calcium physiology related to peanut abiotic stress resistance, it is of vitally important to isolate and characterize more candidate genes for understanding some mechanisms regulating peanut pod development, especially Ca^2+^ and hormone regulating pathway.

With the development of molecular biological techniques, the genomic research has been conducted and made a considerable progress in model legume, e.g., soybean (Severin et al., 2010; Wilson and Grant, 2010; Woody et al., 2011) and *Medicago* (Cannon et al., 2005), but relatively less progress in peanut (Pandey et al., 2012; Zhang et al., 2012). The advent of rapid and high-throughput technology for quantification of the transcriptome (Malone and Oliver, 2011) benefited the peanut genomics research, and was used in the seed development and tissue expression of peanut (Zhang et al., 2012; Chen et al., 2013; Wang et al., 2013; Zhu et al., 2014). These researches make it probability to study the relationship of between calcium and peanut pod development by transcriptome method, and explore valuably candidate genes. In the present study, to better understand the roles of Ca^2+^ in pod development, we compared the transcriptome profile of peanut pod at different developmental stages, through which some potential candidate genes was identified to be related to calcium and hormone.

## Materials and Methods

### Plant Materials and Treatments

Peanut cultivar, ‘Huayu 22,’ was provided by Biotechnology Research Center, Shandong Academy of Agricultural Science (SAAS, China). Free Ca^2+^ content in soil is 14 g (free Ca^2+^) kg^-1^ (dry soil). The pods were collected at the 1st (Stage 1, abbr. S1), 5th (Stage 2, abbr. S2), 10th (Stage 3, abbr. S3), 20th (Stage 4, abbr. S4) day after the peg elongation into the soil. Four stage materials were used for Ca^2+^ excretion determination. The materials of S2, S3, and S4 were collected and immediately frozen in liquid nitrogen, and then stored in a freezer at -80°C for comparative transcriptome analysis.

### Calcium oxide (CaO) Application in Soil

Field experiments was conducted to observe effects of free Ca^2+^ in soil on pod development in Sanzhuang, Rizhao, Shandong Province in spring 2014 and 2015. Free Ca^2+^ content in soil is 4 g (free Ca^2+^) kg^-1^ (dry soil). The 120 kg CaO ha^-1^ was applied in soil as Ca^2+^ treatment, and non-CaO treatment was as control. Each treatment had three replicates. CaO was applied before sowing.

### Ca^2+^ Excretion Measured by Non-invasive Micro-test Technology (NMT)

Ca^2+^ excretion in pods of peanut were measured with NMT system (NMT100 Series, YoungerUSA LLC, Amherst, MA, United States; Xuyue (Beijing) Science and Technology Co., Ltd., Beijing, China) and iFluxes/imFluxes 1.0 (YoungerUSA, LLC, Amherst, MA, United States) Software. Steady-state ion fluxes were measured for 5–20 min. After that, the test treatment was applied and the ion flux in the meristematic zone (120 μm from the root/pod tip) was measured for further 10 min and recorded the data (Yue et al., 2012). And the roots and pods were treated with 40 μm Ca^2+^ solution. Each treatment was repeated at least three times and all tests were repeated at least six times.

### Total RNA Isolation and mRNAs Purification

Total RNA was isolated and integrity confirmed using a 2100 Bioanalyzer (Agilent Technologies). Beads with oligo(dT) were used to isolate poly(A) mRNA from total RNA (Qiagen GmbH, Hilden, Germany).

### Synthesis of cDNA and Sequencing

Following purification, the mRNA was fragmented using divalent cations under elevated temperature. Taking these short fragments as templates, the first-strand cDNA was synthesized using random hexamer primers and Superscript^TM^ III (Invitrogen^TM^, Carlsbad, CA, United States). The second strand cDNA was synthesized using buffer, dNTPs, RNaseH, and DNA polymerase I. Short fragments were purified with a QiaQuick PCR extraction kit (Qiagen) and resolved with EB buffer for end reparation and poly(A) addition. The short fragments were then connected using sequencing adapters. After agarose gel electrophoresis, suitable fragments were used as templates for PCR amplification. Finally, the library was sequenced from both directions on HiSeq 2000 System (illumina, San Diego, CA, United States) with 100 bp of data collected per run by applying TruSeq PE Cluster and TruSeq SBS Kits (illumina, San Diego, CA, United States). Data analysis and base calling were achieved by applying the illumina instrument software.

### Transcriptome *De Novo* Assembly

The reference transcripts of a progenitor of cultivated peanut (*Arachis ipaensis*) were used to generate an integrated reference library. The processing and assembly of transcriptome data were performed with the application of modified Velvet to construct unique consensus sequences (Zerbino and Birney, 2008). The gene expression profile was developed by mapping trimmed transcriptome reads onto the unique consensus sequences by using SOAP2 (Li et al., 2009).

### Annotation and Classification of Unigenes

Comparative transcriptomics analysis was conducted among the three different developmental stages. The intensity values of each sample were further transformed on log_2_-scale and used for differential expression analysis. The probe sets with a *P-*value < 0.01 and > two-fold changes in at least one of the comparisons were considered as differentially expressed genes (DEGs) for further analysis. Unigenes were used for BLAST searches and annotation against an NCBI Nr protein database (NCBI non-redundant sequence database) by using an *E*-value cut-off of 10^-5^ (*E*-value < 0.00001). Unigenes sequences were further aligned by BLASTX to protein databases such as Swiss-Prot, KEGG, and COG, retrieving proteins with the highest sequence similarity with the given unigenes along with their protein functional annotations. If results of different databases conflicted, a priority order of Nr, Swiss-Prot, KEGG, and COG was followed. For unigenes that did not align to any of the above databases, EST Scan software (Iseli et al., 1999) was used to predict their coding regions and determine sequence direction. Unigenes aligned to databases with higher priority did not enter the next circle. The alignments were considered complete when all circles were finished. The coding region sequences were then determined for proteins with the highest ranks using BLAST. Unigenes that could not be aligned to any database were scanned by EST Scan (Iseli et al., 1999) to determine the nucleotide (50–30) and amino acid sequences of the coding regions. The Blast2 GO was used to obtain Gene Ontology (GO) annotations for the unigenes, as well as for KEGG and COG analysis (Conesa et al., 2005). The WEGO software was then used to perform GO functional classification of all unigenes to view the distribution of gene functions of the species at the macro level (Ye et al., 2006). It was mapped all of the annotated unigenes to GO terms in the database and calculated the number of unigenes associated with every term.

### Real-Time Fluorescent Quantitative PCR Analysis

Total RNA was isolated and purified using TRIzol Reagent (Tiangen, China) according to the manufacture’s instruction. The first-strand cDNA was synthesized by using Reverse Transcriptase M-MLV (Takara, Japan) according to the manufacturer’s protocol. The PCR was amplified following the instruction of SYBR *Premix Ex Taq*^TM^ (Takara, Japan) with the fluorescent quantitative PCR amplification instrument (ABI 7500, United States). The target gene primers were used to detect the sample mRNA. Control reactions were carried out by using primers Tua5-F and Tua5-R to detect the transcript encoding ubiquitously expressed tua5 (Chi et al., 2012). All assays for a target gene were performed in triplicate synchronously under identical conditions.

## Results

### Effects of Calcium on Peanut Pod Development and Ca^2+^ Excretion of Different Pod Tissues

**FIGURE 1:**
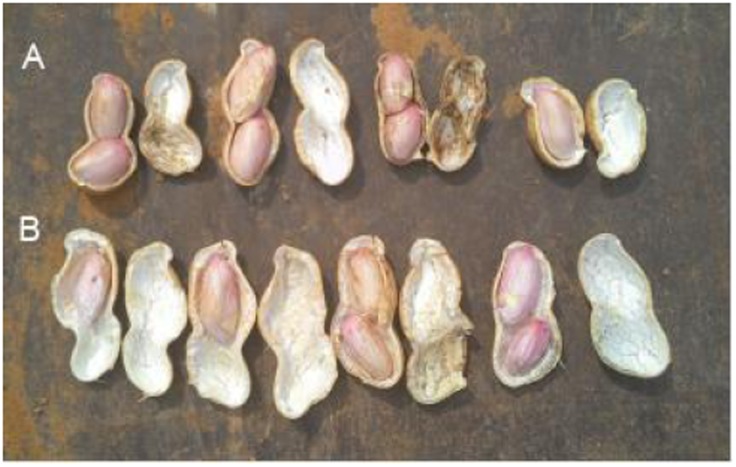
Effects of soil free Ca^2+^ on peanut pod development. The experiments were conducted on a farmland with 4 g (free Ca^2+^) kg^-1^ (dry soil). **(A)** Applied 120 kg CaO ha^-1^. **(B)** Without CaO application.

Peanut is one of the important oil crops for China, and its yield is often affected by all kinds of environmental factors, including free Ca^2+^ content in soil. When free Ca^2+^ is deficient in soil, it will cause unfilling of pods. **Figure 1** showed that pods were unfilled under 4 g (free Ca^2+^) kg^-1^ (dry soil), and CaO application can benefit pod filling.

**FIGURE 2:**
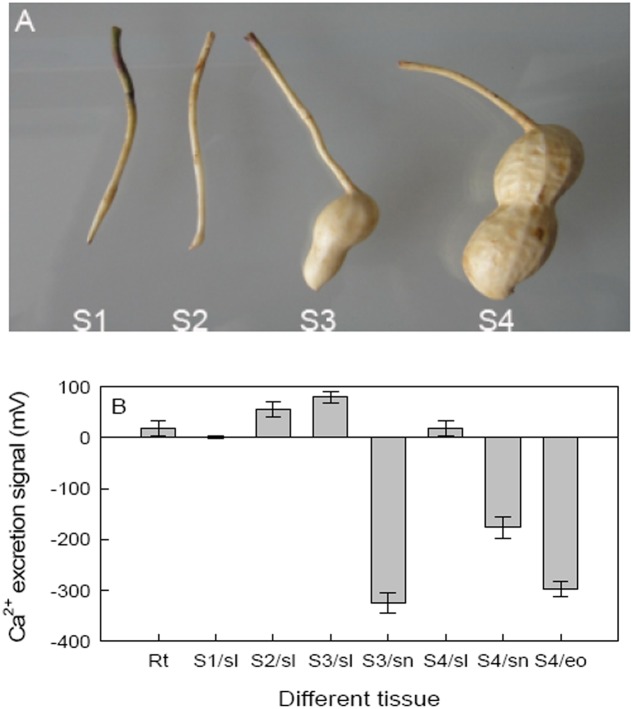
Effects of calcium on peanut pod development and the Ca^2+^ excretion of different pod tissues. **(A)** Hair roots and four different pod developing stages were selected as detecting materials. **(B)** Ca^2+^ excretion was further detected on different tissues of these stages. Each value was averaged from more than three replicates (mean ± SD).

To assess the role of Ca^2+^ on pod development, hair root and four stages of pods were analyzed to determine Ca^2+^ excretion where: the peg just entered the soil (S1), tip of the peg initiated expansion (S2), beginning of pod reticulation (S3), and when the pod was clearly reticulated (S4), respectively, used to analyze (**Figure 2A**). The non-invasive microtest technique was used to detect net fluxes of Ca^2+^ at different seed tissues of different stages. Besides hair roots, peanut shells showed Ca^2+^ excretion at all four stages; however, both the embryo of S4 and the red skin of S3 and S4 showed Ca^2+^ absorbance. These results showed that embryo alone and embryo with red skin of peanut need Ca^2+^ during development (**Figure 2B**).

### Assembly and Functional Annotations of Peanut Seed Transcriptomes

**FIGURE 3:**
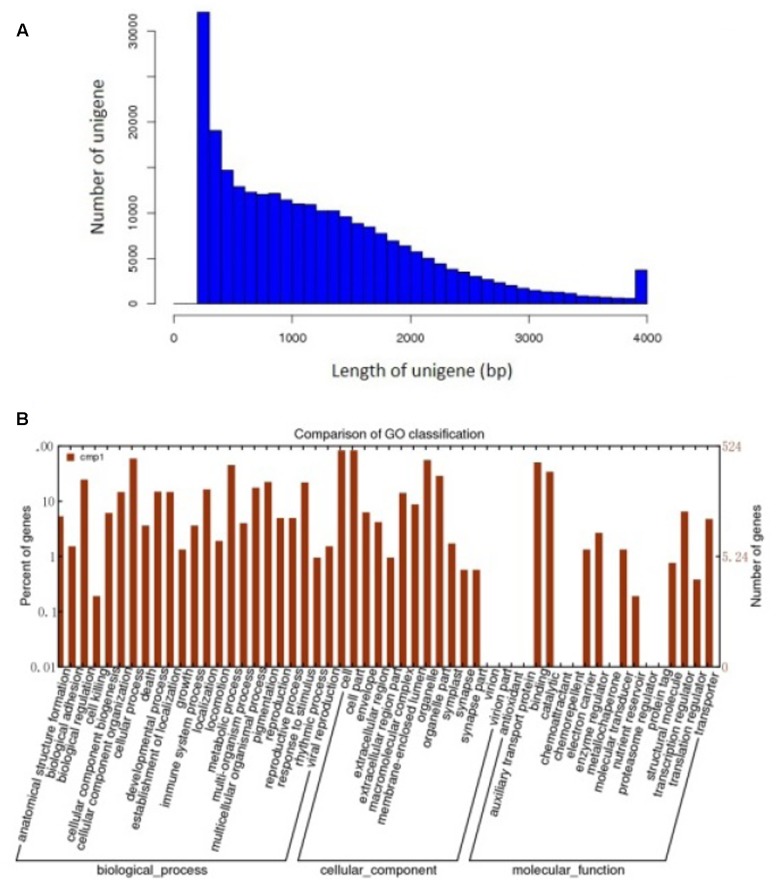
Assembly and functional annotations of peanut seed transcriptomes. **(A)** Sequence lengths of 263,110 assembled peanut unigenes. **(B)** A histogram of unigene ontology classification.

To further detect some mechanisms relate to the roles of Ca^2+^ in pod development. About 70 million high quality reads were obtained from the library. The average read size, Q30 percentage (sequencing error rate, 0.1%), and GC percentage for each library was 90 bp, >95%, and >40%, respectively. Clean reads from each library were used for assembly separately. And 263,110 unigenes were generated from library. The *de novo* assembly of all sequencing data using the modified Velvet was followed by the application of the SOAP2 program for unigene identification. The average sequence length of the unigenes was 1,235 bp and its range was 201–4,000 bp (**Figure 3A**).

The GO assignments were used to categorize the functions of the predicted peanut Unigenes. Based on the sequence homologies, the sequences were categorized into 45 functional groups (**Figure 3B**), contained three main categories of the GO classification (biological processes, cellular components, and molecular functions). The genes involved in cellular process and metabolic process were dominant in the “Biological Process” category. “Cell,” “Cell part,” and the “Organelle” are the top three abundant categories in “Cellular component,” While “Binding,” and “catalytic activity” are dominant in the “Molecular function” category (**Figure 3B**).

### Comparative Analysis of Three Pod Developmental Stages

**FIGURE 4:**
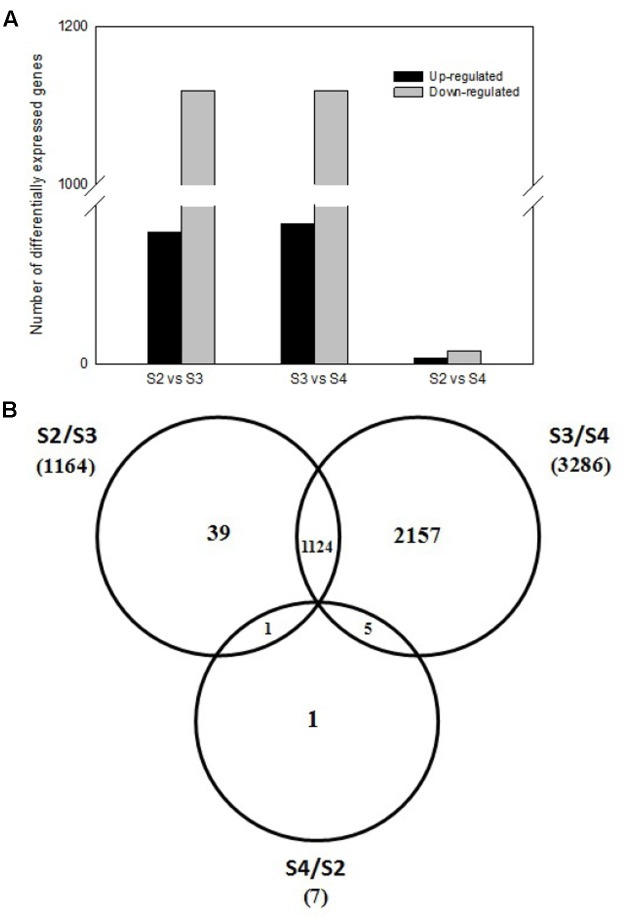
Comparative analysis of three pod developmental stages. **(A)** Comparative analysis of different expressed genes between three pod developmental stages. **(B)** The number of differentially expressed genes between three pods development stages. The numbers of up-regulated and down-regulated genes are indicated at the 5th (S2), 10th (S3), 20th (S4) day after the peg elongation into the soil.

To investigate expression levels of stage-specific genes during pods development, we conducted comparative analysis of the transcriptome profiles among three pod development stages at 5th (S2), 10th (S3), 20th (S4) day after the peg elongation into the soil. As shown in **Figure 4A**, totally 1,169 DEGs were obtained between S2 and S3, including 50 up-regulated and 1,119 down-regulated genes. Similarly, 1,172 DEGs (including 53 up-regulated and 1,119 down-regulated genes), 7 DEGs (including two up-regulated and five down-regulated genes) were identified between S3 and S4, S2 and S4, respectively. In addition, some other stage-specific expression genes were also analyzed. Approximately 39, 2157, and 1 stage-specific expression genes were identified between S2 and S3, S3 and S4, S2 and S4, respectively (**Figure 4B**).

### Calcium and Ca^2+^ Signal Related Unigenes and Their Functional Categories

Since deficient free Ca^2+^ in soil can cause unfilling of pods, some DEGs involved in biological process of Ca^2+^ binding and Ca^2+^ signaling were identified based on GO analysis. These potential candidate genes contained 53 Ca^2+^ or Ca^2+^ signal pathway related genes (**Table 1**). These genes were significant differentially expressed during seed and pod development, suggesting that Ca^2+^ and calcium related protein might be involved in seed development.

**Table 1:** The annotation of candidate genes related to calcium during pods development.

Gene ID	Uniprot No.	Gene description	Species	*E*-value
comp33933	Q8RYJ8	Putative calcium-binding protein CML19	*Oryza*	4.00*e* - 23
comp43665	Q9LE22	Probable calcium-binding protein CML27	*Arabidopsis*	5.00*e* - 55
comp36255	Q8L3R2	Probable calcium-binding protein CML41	*Arabidopsis*	9.00*e* - 37
comp50658	Q9ZQH1	Probable calcium-binding protein CML48	*Arabidopsis*	1.00*e* - 13
comp53390	Q9LF79	Calcium-transporting ATPase 8	*Arabidopsis*	0
comp51237	Q9FKP1	Cation/calcium exchanger 1	*Arabidopsis*	3.00*e* - 174
comp45295	Q9FKP2	Cation/calcium exchanger 2	*Arabidopsis*	1.00*e* - 50
comp14601	Q8LEM7	Calcineurin B-like protein 3	*Arabidopsis*	1.00*e* - 63
comp47042	O81223	Calcineurin B-like protein 4	*Arabidopsis*	8.00*e* - 100
comp47926	O65718	Cyclic nucleotide-gated ion channel 2	*Arabidopsis*	0
comp42319	P51074	Annexin-like protein RJ4	*Fragaria*	2.00*e* - 158
comp45239	Q940H6	Serine/threonine-protein kinase SRK2E	*Arabidopsis*	0
comp36540	Q96262	Plasma membrane-associated cation-binding protein 1	*Arabidopsis*	1.00*e* - 47
comp48463	Q93ZH2	Nuclear transcription factor Y subunit A-3	*Arabidopsis*	3.00*e* - 36
comp40687	P34913	Bifunctional epoxide hydrolase 2	*Homo*	2.00*e* - 19
comp49372	Q8LFG1	Probable alpha-amylase 2	*Arabidopsis*	1.00*e* - 149
comp52512	P14226	Oxygen-evolving enhancer protein 1	*Pisum*	0
comp38946	P16059	Oxygen-evolving enhancer protein 2	*Pisum*	2.00*e* - 151
comp46793	Q41932	Oxygen-evolving enhancer protein 3-2	*Arabidopsis*	3.00*e* - 81
comp48726	Q39254	Vacuolar cation/proton exchanger 2	*Arabidopsis*	8.00*e* - 6
comp45043	Q93Z81	Vacuolar cation/proton exchanger 3	*Arabidopsis*	2.00*e* - 151
comp47437	Q2HXL0	Respiratory burst oxidase homolog protein C	*Solanum*	0
comp51314	O48538	Respiratory burst oxidase homolog protein F	*Arabidopsis*	0
comp31869	Q39033	Phosphoinositide phospholipase C 2	*Arabidopsis*	8.00*e* - 50
comp50062	P17859	Phosphoinositide phospholipase C 2	*Arabidopsis*	8.00*e* - 50
comp41809	O82598	Aquaporin TIP1-3	*Arabidopsis*	2.00*e* - 129
comp33153	A5A7I7	Calcium-dependent protein kinase 4	*Solanum*	0
comp45958	Q9SSF8	Calcium-dependent protein kinase 30	*Arabidopsis*	0
comp44027	P28583	Calcium-dependent protein kinase SK5	*Glycine*	0
comp52882	Q6DN14	Multiple C2 and transmembrane domain-containing protein 1	*Homo*	2.00*e* - 10
comp50090	Q6DN12	Multiple C2 and transmembrane domain-containing protein 2	*Homo*	4.00*e* - 23
comp41514	O81270	Peroxygenase 1	*Arabidopsis*	1.00*e* - 107
comp51310	Q641Z6	EH domain-containing protein 1	*Rattus*	2.00*e* - 146
comp53583	Q8L493	Branched-chain-amino-acid aminotransferase-like protein 3	*Arabidopsis*	6.00*e* - 134
comp46176	O81916	Uncharacterized calcium-binding protein At1g02270	*Arabidopsis*	1.00*e* - 150
comp43596	Q8LBL1	Two-pore potassium channel 1	*Arabidopsis*	2.00*e* - 131
comp40656	Q9LDQ3	Putative cysteine-rich receptor-like protein kinase 35	*Arabidopsis*	6.00*e* - 78
comp52241	Q8L7E9	Protein MID1-COMPLEMENTING ACTIVITY 1	*Arabidopsis*	0
comp52082	Q94A4	Alpha-amylase 3, chloroplastic	*Arabidopsis*	0
comp52718	Q9C8E7	Glutamate receptor 3.3	*Arabidopsis*	0
comp43681	Q9LSQ6	Calcium-binding protein PBP1	*Arabidopsis*	4.00*e* - 37
comp34785	Q9LSQ6	Calcium-binding protein PBP1	*Arabidopsis*	3.00*e* - 43
comp49728	Q41142	Phospholipase D alpha 1	*Ricinus*	2.00*e* - 43
comp50171	Q9FYH7	Vacuolar-sorting receptor 6	*Arabidopsis*	4.00*e* - 13
comp50757	Q9FYH7	Vacuolar-sorting receptor 6	*Arabidopsis*	0
comp44749	Q93Z81	Vacuolar cation/proton exchanger 3	*Arabidopsis*	5.00*e* - 171
comp46345	P42055	Mitochondrial outer membrane protein porin of 34 kDa	*Solanum*	1.00*e* - 35
comp51265	Q9C9L5	Wall-associated receptor kinase-like 9	*Arabidopsis*	5.00*e* - 8
comp25145	F4JJJ3	NAD(P)H dehydrogenase B3, mitochondrial	*Arabidopsis*	0
comp48262	Q9BVG8	Kinesin-like protein KIFC3	*Homo*	4.00*e* - 60
comp49233	Q9FKI0	Fimbrin-like protein 2	*Arabidopsis*	0
comp45947	Q298L5	Mitochondrial Rho GTPase	*Sophophora*	5.00*e* - 87
comp42934	Q9S7C9	Putative DNA-binding protein ESCAROLA	*Arabidopsis*	5.00*e* - 6


### Hormone Related Unigenes and Their Functional Categories

Genes related to hormone response were screened out by GO analysis. There were 40 auxin related genes, 15 gibberellin related genes, 20 ethylene related genes, 2 abscisic acid related genes, and 7 cytokinin related genes (**Table 2**) showed significant differentially expression during seed and pod development.

**Table 2:** The annotation of candidate genes related to hormone during pods development.

Gene ID	Uniprot No.	Gene description	Species	*E*-value
**Auxin-related**				
comp43478	Q6J163	Auxin-induced protein 5NG4	*Pinus*	4.00*e* - 8
comp47743	Q6J163	Auxin-induced protein 5NG4	*Pinus*	1.00*e* - 49
comp39204	Q6J163	Auxin-induced protein 5NG4	*Pinus*	3.00*e* - 15
comp51971	Q6J163	Auxin-induced protein 5NG4	*Pinus*	2.00*e* - 46
comp48589	Q6J163	Auxin-induced protein 5NG4	*Pinus*	6.00*e* - 77
comp44220	Q6J163	Auxin-induced protein 5NG4	*Pinus*	1.00*e* - 39
comp40373	Q6J163	Auxin-induced protein 5NG4	*Pinus*	5.00*e* - 29
comp48904	Q6J163	Auxin-induced protein 5NG4	*Pinus*	2.00*e* - 11
comp39544	Q6J163	Auxin-induced protein 5NG4	*Pinus*	1.00*e* - 42
comp44395	Q6J163	Auxin-induced protein 5NG4	*Pinus*	1.00*e* - 29
comp45206	Q6J163	Auxin-induced protein 5NG4	*Pinus*	2.00*e* - 56
comp49555	Q6J163	Auxin-induced protein 5NG4	*Pinus*	3.00*e* - 29
comp41016	Q6J163	Auxin-induced protein 5NG4	*Pinus*	2.00*e* - 64
comp43783	Q6J163	Auxin-induced protein 5NG4	*Pinus*	8.00*e* - 6
comp41488	Q6J163	Auxin-induced protein 5NG4	*Pinus*	5.00*e* - 47
comp46020	Q6J163	Auxin-induced protein 5NG4	*Pinus*	3.00*e* - 20
comp47084	Q6J163	Auxin-induced protein 5NG4	*Pinus*	1.00*e* - 28
comp34759	Q6J163	Auxin-induced protein 5NG4	*Pinus*	2.00*e* - 62
comp35338	Q6J163	Auxin-induced protein 5NG4	*Pinus*	5.00*e* - 7
comp45159	Q6J163	Auxin-induced protein 5NG4	*Pinus*	3.00*e* - 81
comp36946	Q6J163	Auxin-induced protein 5NG4	*Pinus*	1.00*e* - 129
comp43310	Q6J163	Auxin-induced protein 5NG4	*Pinus*	3.00*e* - 146
comp43748	Q8LAL2	Auxin-responsive protein IAA26	*Arabidopsis*	1.00*e* - 70
comp36524	Q9ZSY8	Auxin-responsive protein IAA27	*Arabidopsis*	8.00*e* - 91
comp35718	Q8H174	Auxin-responsive protein IAA31	*Arabidopsis*	3.00*e* - 31
comp50484	Q94BT2	Auxin-induced in root cultures protein 12	*Arabidopsis*	3.00*e* - 6
comp46184	Q94BT2	Auxin-induced in root cultures protein 12	*Arabidopsis*	8.00*e* - 15
comp44924	Q94BT2	Auxin-induced in root cultures protein 12	*Arabidopsis*	2.00*e* - 15
comp35357	Q9LF79	Auxin-induced protein 15A	*Glycine*	2.00*e* - 19
comp30777	P33081	Auxin-induced protein 15A	*Glycine*	1.00*e* - 20
comp36019	P33079	Auxin-induced protein 10A5	*Glycine*	8.00*e* - 13
comp36785	Q9FEL6	Auxin transporter-like protein 3	*Medicago*	0
comp45084	O24543	Auxin-induced protein 22E	*Vigna*	1.00*e* - 63
comp52994	Q9FGV1	Auxin response factor 8	*Arabidopsis*	7.00*e* - 133
comp46658	Q9XED8	Auxin response factor 9	*Arabidopsis*	0
comp28367	Q653H7	Auxin response factor 18	*Oryza*	7.00*e* - 63
comp45289	Q653H7	Auxin response factor 18	*Oryza*	1.00*e* - 162
comp33145	Q653H7	Auxin response factor 18	*Oryza*	7.00*e* - 47
comp32944	Q96247	Auxin transporter protein 1	*Arabidopsis*	9.00*e* - 163
comp41401	O04012	Auxin-binding protein ABP19	*Prunus*	5.00*e* - 74
**Gibberellin-related**				
comp53643	O04706	Gibberellin 20 oxidase 1-B	*Triticum*	1.00*e* - 121
comp46516	Q39111	Gibberellin 20 oxidase 2	*Arabidopsis*	2.00*e* - 129
comp44953	Q39111	Gibberellin 20 oxidase 2	*Arabidopsis*	8.00*e* - 80
comp45036	Q9SQ80	Gibberellin 2-beta-dioxygenase 1	*Pisum*	4.00*e* - 160
comp48090	Q9SQ80	Gibberellin 2-beta-dioxygenase 1	*Pisum*	6.00*e* - 162
comp43715	Q9XFR9	Gibberellin 2-beta-dioxygenase 2	*Arabidopsis*	6.00*e* - 17
comp47582	O49561	Gibberellin 2-beta-dioxygenase 8	*Arabidopsis*	4.00*e* - 46
comp40312	O49561	Gibberellin 2-beta-dioxygenase 8	*Arabidopsis*	3.00*e* - 51
comp44039	Q39103	Gibberellin 3-beta-dioxygenase 1	*Arabidopsis*	1.00*e* - 88
comp45509	Q9SVS8	Gibberellin 3-beta-dioxygenase 3	*Arabidopsis*	3.00*e* - 20
comp37867	P46690	Gibberellin-regulated protein 4	*Arabidopsis*	6.00*e* - 38
comp41469	P46690	Gibberellin-regulated protein 4	*Arabidopsis*	7.00*e* - 33
comp13386	Q9LFR3	Gibberellin-regulated protein 14	*Arabidopsis*	1.00*e* - 19
comp27867	F4IQJ4	Gibberellin-regulated protein 11	*Arabidopsis*	1.00*e* - 23
comp40799	Q9FDW1	Transcription factor MYB44	*Arabidopsis*	3.00*e* - 32
**Ethylene-related**				
comp35300	Q84QC2	Ethylene-responsive transcription factor ERF017	*Arabidopsis*	7.00*e* - 33
comp11865	Q8LBQ7	Ethylene-responsive transcription factor ERF034	*Arabidopsis*	9.00*e* - 23
comp45116	Q9SKT1	Ethylene-responsive transcription factor ERF053	*Arabidopsis*	5.00*e* - 43
comp34495	Q9LY05	Ethylene-responsive transcription factor ERF106	*Arabidopsis*	5.00*e* - 35
comp37722	Q9CA27	Ethylene-responsive transcription factor ERF118	*Arabidopsis*	5.00*e* - 20
comp53691	Q8LC30	Ethylene-responsive transcription factor RAP2-1	*Arabidopsis*	9.00*e* - 41
comp42029	P42736	Ethylene-responsive transcription factor RAP2-3	*Arabidopsis*	2.00*e* - 37
comp48452	Q6X5Y6	Ethylene-responsive transcription factor WRI1	*Arabidopsis*	3.00*e* - 37
comp44242	O80340	Ethylene-responsive transcription factor 4	*Arabidopsis*	9.00*e* - 9
comp42489	Q40477	Ethylene-responsive transcription factor 4	*Nicotiana*	2.00*e* - 22
comp37530	Q8L9K1	Ethylene-responsive transcription factor 13	*Arabidopsis*	2.00*e* - 34
comp47316	Q9SUQ2	Ethylene-responsive transcription factor CRF2	*Arabidopsis*	1.00*e* - 33
comp43675	Q9SUE3	Ethylene-responsive transcription factor CRF4	*Arabidopsis*	4.00*e* - 50
comp47833	Q9LVG2	AP2-like ethylene-responsive transcription factor TOE2	*Arabidopsis*	1.00*e* - 6
comp43442	Q38914	AP2-like ethylene-responsive transcription factor ANT	*Arabidopsis*	1.00*e* - 132
comp51344	Q38914	AP2-like ethylene-responsive transcription factor ANT	*Arabidopsis*	2.00*e* - 19
comp48832	Q1PFE1	AP2-like ethylene-responsive transcription factor AIL1	*Arabidopsis*	4.00*e* - 129
comp47962	Q52QU2	AP2-like ethylene-responsive transcription factor AIL6	*Arabidopsis*	5.00*e* - 14
comp50155	Q0WPQ2	Ethylene receptor 2	*Arabidopsis*	0
comp42987	Q8GWK2	AP2-like ethylene-responsive transcription factor At2g41710	*Arabidopsis*	2.00*e* - 148
**Cytokinin-related**				
comp44683	Q9ZW95	Cytokinin hydroxylase	*Arabidopsis*	6.00*e* - 72
comp14370	Q9FF18	Cytokinin hydroxylase	*Arabidopsis*	22.00*e* - 516
comp42133	Q9FF18	Cytokinin hydroxylase	*Arabidopsis*	2.00*e* - 175
comp42347	Q9FUJ1	Cytokinin dehydrogenase 7	*Arabidopsis*	3.00*e* - 41
comp40332	Q9FUJ1	Cytokinin dehydrogenase 7	*Arabidopsis*	1.00*e* - 166
comp53620	Q8L8B8	Cytokinin riboside 5’-monophosphate phosphoribohydrolase LOG3	*Arabidopsis*	4.00*e* - 130
comp38806	Q84MC2	Cytokinin riboside 5’-monophosphate phosphoribohydrolase LOG8	*Arabidopsis*	2.00*e* - 127
**Abscisic acid-related**				
comp38442	Q8RYD6	Abscisic acid insensitive 5-like protein 1	*Arabidopsis*	3.00*e* - 28
comp29840	Q9FH76	Abscisic acid 8’-hydroxylase 3	*Arabidopsis*	5.00*e* - 160


### Real-Time Fluorescent Quantitative PCR Analysis

**FIGURE 5:**
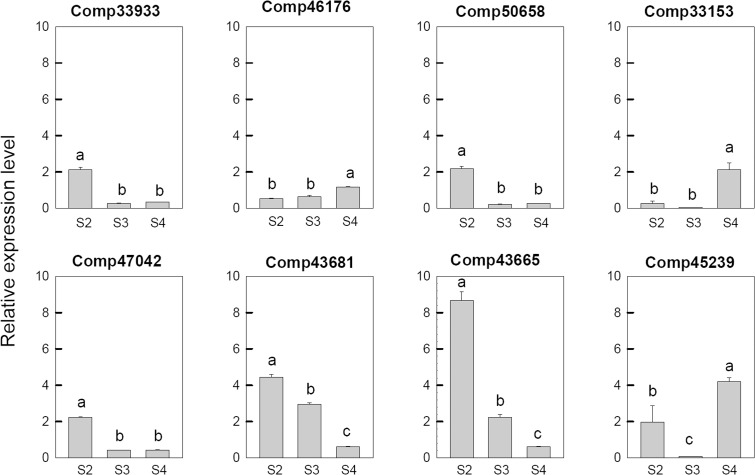
Real-time fluorescent quantitative PCR analysis on mRNA transcription of the selected differentially expressed calcium related genes. These were the 5th (S2), 10th (S3), 20th (S4) day after the peg elongation into the soil. The ordinate axis greater than one means up-regulated, less than one was down-regulated.

In order to confirm the transcriptome sequencing results, Ca^2+^ and hormone related genes were selected from DEGs based on the GO analysis and used to real-time fluorescent quantitative PCR analysis. As shown in **Figure 5**, the expression pattern of 8 selected DEGs, which are related to Ca^2+^ signal pathway, were consistent with their respective microarrays data. Among these genes, two genes, which includes one CDPK gene (comp33153) and one uncharacterized calcium-binding protein At1g02270 (comp46176), were up-regulated at S4. The Serine/threonine-protein kinase SRK2E (comp45239) was up-regulated at S2, down-regulated at S3, and then up-regulated at S4. Three probable calmodulin like protein CMLs (comp33933, comp43665, and comp50658), one calcineurin B-like protein (comp47042) and one calcium-binding protein PBP1 (comp43681) were up-regulated at S2 and then down-regulated during the later stages.

**FIGURE 6:**
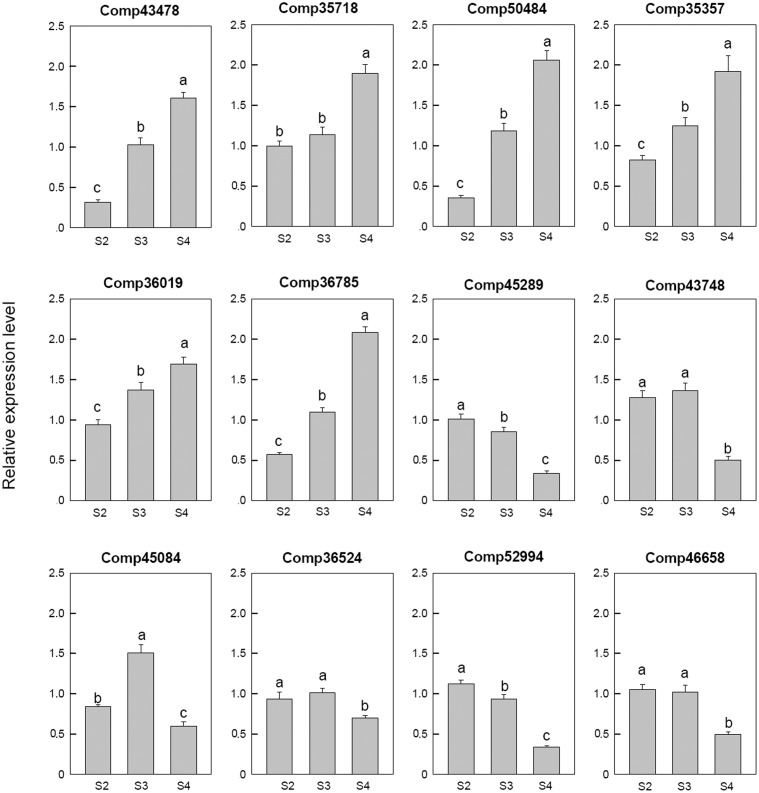
Real-time fluorescent quantitative PCR analysis on mRNA transcription of the selected differentially expressed auxin related genes. These were the 5th (S2), 10th (S3), 20th (S4) day after the peg elongation into the soil. The ordinate axis greater than one means up-regulated, less than one was down-regulated.

Among the 12 selected auxin related genes, six genes, i.e., auxin-induced protein 5NG4 (comp43478), auxin-responsive protein IAA31 (comp35718), auxin-induced in root cultures protein 12 (comp50484), auxin-induced protein 15A (comp35357), auxin-induced protein 10A5 (comp36019), and auxin transporter-like protein 3 (comp36785), were down-regulated in S2 and then up-regulated during the later stages. Three auxin response factor 8 (comp52994, comp45289, and comp46658), one auxin-responsive protein IAA26 (comp36524) were down-regulated at S4. One auxin-induced protein 22E (comp45084) was down-regulated at S2, up-regulated at S3, and then down-regulated at S4 (**Figure 6**).

**FIGURE 7:**
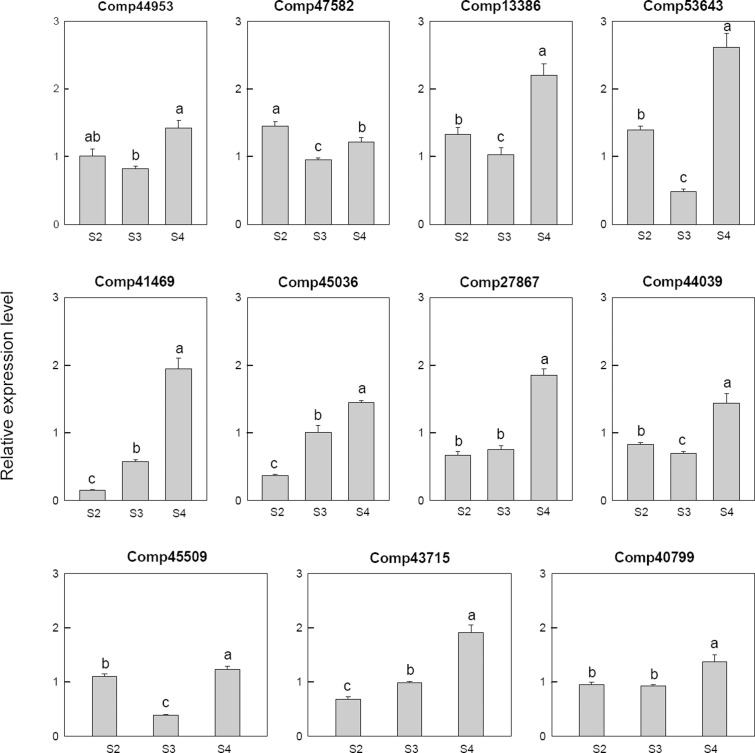
Real-time fluorescent quantitative PCR analysis on mRNA transcription of the selected differentially expressed gibberellin related genes. These were the 5th (S2), 10th (S3), 20th (S4) day after the peg elongation into the soil. The ordinate axis greater than one means up-regulated, less than one was down-regulated.

Among 11 selected gibberellin related genes, four genes, i.e., two gibberellin-regulated protein (comp41469, comp27867), two gibberellin 2-beta-dioxygenase (comp45036, comp43715), were down-regulated at S2 and then up-regulated at S4. One transcription factor MYB44 (comp40799), one gibberellin 20 oxidase (comp44953) and one gibberellin 3-beta-dioxygenase (comp44039) were up-regulated at S4. One gibberellin 20 oxidases (comp53643), one gibberellin 2-beta-dioxygenase (comp47582), one gibberellin 3-beta-dioxygenase (comp45509) and one gibberellin-regulated protein (comp13386) were up-regulated at S2, down-regulated at S3 and then up-regulated at S4 (**Figure 7**).

**FIGURE 8:**
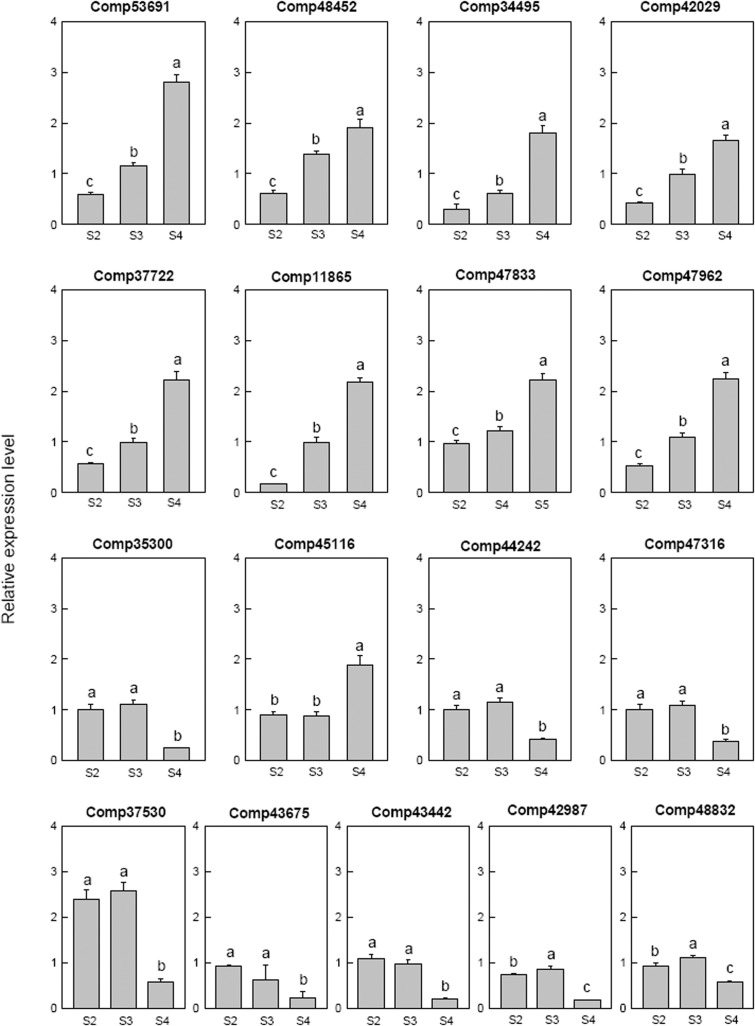
Real-time fluorescent quantitative PCR analysis on mRNA transcription of the selected differentially expressed ethylene related genes. These were the 5th (S2), 10th (S3), 20th (S4) day after the peg elongation into the soil. The ordinate axis greater than one means up-regulated, less than one was down-regulated.

Seventeen selected ethylene related genes were analyzed. Two ethylene-responsive transcription factor RAP2 (comp53691 and comp42029), one ethylene-responsive transcription factor WRI1 (comp48452), three ethylene-responsive transcription factor ERF (comp34495, comp37722, and comp11865) and one AP2-like ethylene-responsive transcription factor AIL (comp47962) were down-regulated at S2 then up-regulated at S4. One ethylene-responsive transcription factor ERF (comp35300), one ethylene-responsive transcription factor (comp44242), two ethylene-responsive transcription factor CRF (comp47316 and comp43675) and one AP2-like ethylene-responsive transcription factor ANT (comp43442) were only down-regulated at S4. The AP2-like ethylene-responsive transcription factor TOE (comp47833) and ethylene-responsive transcription factor ERF (comp45116) were up-regulated at S4. AP2-like ethylene-responsive transcription factor At2g41710 (comp42987) and AP2-like ethylene-responsive transcription factor AIL1 (comp48832) were down-regulated at S2, up-regulated at S3, and then down-regulated at S4. The ethylene-responsive transcription factor 13 (comp37530) was up-regulated at S2 and S3, then down-regulated at S4 (**Figure 8**).

**FIGURE 9:**
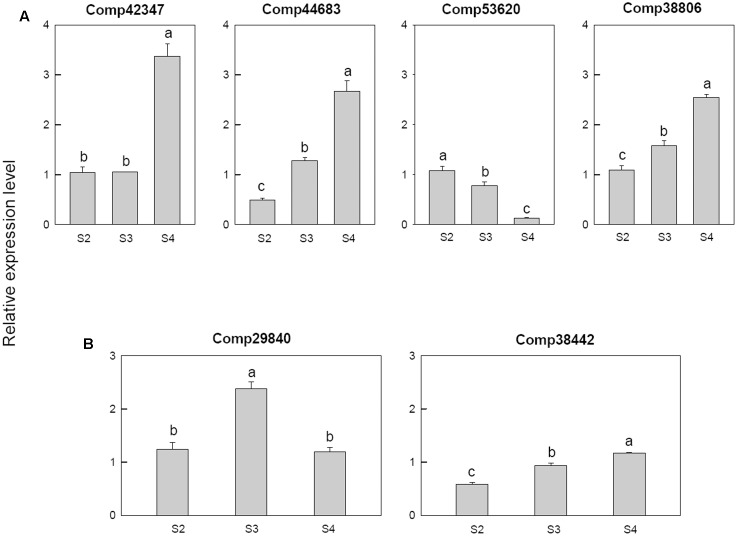
Real-time fluorescent quantitative PCR analysis on mRNA transcription of the selected differentially expressed genes. **(A)** Cytokinin related genes, **(B)** abscisic acid related genes. These were the 5th (S2), 10th (S3), 20th (S4) day after the peg elongation into the soil. The ordinate axis greater than one means up-regulated, less than one was down-regulated.

Among the four cytokinin related genes, cytokinin dehydrogenase (comp42347) was only up regulated at S4, cytokinin hydroxylase (comp44683) was down-regulated at S2 then up-regulated in later stage, cytokinin riboside 5′-monophosphate phosphoribohydrolase LOG3 (comp53620) was down-regulated from S3 and the Cytokinin riboside 5′-monophosphate phosphoribohydrolase LOG8 (comp38806) up-regulated from S3 (**Figure 9A**).

For the two abscisic acid related genes, the abscisic acid 8′-hydroxylase 3 (comp29840) was up-regulated at S3 and then down-regulated, however, the abscisic acid insensitive 5-like protein 1 (comp38442) was down-regulated at S2 (**Figure 9B**).

## Discussion

Calcium is not only an essential macroelement for plant growth, but also a second messenger involved in regulating diverse physiological processes (Poovaih and Ready, 1993). Peanut remains one of the most important oil-crops in the world, and Calcium is by far the most critical nutrient for this crop to achieve high yields. Calcium deficiency in soil can results in a decrease in yield, e.g., unfilled pods (Cox et al., 1982). Unfilled pods were also found in free Ca^2+^-insufficient soil in the present work (**Figure 1**), and the pod yield would decreased by 20% or more. For pods, it seems that embryo alone or embryo with red skin are the main tissues need more Ca^2+^ (**Figure 2B**). It attracts us to pay more attention to investigate the mechanisms of how Ca^2+^ is involved in pod development.

As a powerful technique, transcriptome sequencing was used to observe global gene expression profiles and the physiological processes involved in various plants (Clarke and Zhu, 2006). As to fruit development, it has been applied to strawberry (Aharoni and O’Connell, 2002), tomato (Alba et al., 2005), pear (Fonseca et al., 2004), apple (Lee et al., 2007), and peanut seed development (Zhang et al., 2012; Zhu et al., 2014). But the relationship between calcium and peanut seed at different pods developmental stages was seldom mentioned. In the present work, 263,110 unigenes were obtained from library. We identified 4,457 DEGs via sequencing analysis, and got 2,197 stage-specific expressed genes at different pod developmental stages (**Figure 4B**). It has been reported that free Ca^2+^ concentration in soil seriously affected peanut fruiting and yield, ovary handle and pods could absorb Ca^2+^ from soil, and transport it to both the developing seed and the vegetative organs, such as stem, leaves, etc. (Beringer and Taha, 1976). In this study, through Gene Ontology analysis, many DEGs involved in the Ca^2+^ responding and Ca^2+^ signal transduction, which were identified as potential candidate genes responsible for development of pods (**Table 1**). That is to say, calcium was related to pod development at the genetic levels.

Besides an important essential element, Ca^2+^ also acts as a secondary messenger in various biological processes. It has been shown that various Ca^2+^ transporters in plants (Dayod et al., 2010) and Ca^2+^ signal transduction protein were involved in gametogenesis, fertilization and early pollen tube growth (Tirlapur and Cresti, 1992). Interestingly, in the present study, we found some calcium-related unigenes at different pod developmental stages, and eight Ca^2+^ signaling-related genes were analyzed. These genes were calcium-dependent protein kinase (CDPK), calcineurin B-like protein (CBL), calmodulin like protein (CML), and some other Ca^2+^ signaling related genes. CDPKs were thought be involved in embryogenesis, seed development and germination of sandalwood (Anil et al., 2000) and in rice seed development (Morello et al., 2000). OsCDPK2 protein had a low level during early seed development, but had a high level which maintained in 20 days after fertilization (Frattini et al., 1999). Overexpression of the CDPK OsCDPK2 in transgenic rice disrupted seed development (Laura et al., 2000). In peanut, CDPK genes might be involved in pod development since they were up-regulated at 20th day after the peg elongation into the soil (**Figure 5**). However, some other calcium signal related genes, such as CBL, CML, and PBP1 were all down-regulated. These results implied that Ca^2+^-signaling transduction pathway might be involved in pod development.

Auxin, gibberellin, abscisic acid, cytokinin, and ethylene have been implicated in regulating the peanut seed development and pod maturation (Jacobs, 1951; Ziv and Kahana, 1988; Shlamovitz et al., 1995; Moctezuma and Feldman, 1996; Ozga and Reinecke, 2003). Meanwhile, genes encoding key enzymes related to hormone metabolism showed significant changes in transcriptional level during peanut early pod development (Xia et al., 2013). In this study, a number of DEGs related to hormone response were identified at different pods developmental stages, e.g., auxin response factor, auxin-induced protein, auxin response factor, gibberellin-regulated protein, gibberellin 2-beta-dioxygenase, gibberellin 3-beta-dioxygenase, ethylene-responsive transcription factor, AP2-like ethylene-responsive transcription factor, cytokinin hydroxylase, cytokinin dehydrogenase, abscisic acid insensitive 5-like protein, and abscisic acid 8′-hydroxylase (**Table 2**). All these revealed that hormone response genes potentially played a crucial role in peanut seed development.

Seed development is a complex process, many physiological processes occurred during seed development (**Figure 3**), calcium and hormone seems to play important roles in this progress, respectively. It has been known that hormonal controls on cell division and expansion are active in the development of fruit. Many of phytohormonal pathways utilize changes in cytoplasmic calcium concentration ([Ca2C]cyt) as a secondary signal messenger (e.g., ABA, JA, auxin, GA, ethylene, brassinosteroids, and cytokinins) (Fortes et al., 2015). Recently, more and more reports showed that Ca^2+^ signal transduction pathway is involved in phytohormonal pathways. In Arabidopsis, a CPK4 is involved in modulating ABA signaling (Wang et al., 2017), meanwhile, CPK28 plays a role in balancing the phytohormones JA and GA in development (Matschi et al., 2015), and affects ethylene biosynthesis (Jin et al., 2017). Additionally, the calmodulin-like protein CML20, a functional Ca^2+^ sensor, negatively regulates ABA-induced stomatal movement in Arabidopsis (Wu et al., 2017), StCDPK3 in involved in the cross-talk between ABA and GA signaling at the onset of potato tuber development (Grandellis et al., 2016).

The present study provided basis for finding and screening calcium related gene and hormone response genes during peanut seed development. We speculated that besides being components of cell, such as membrane or something else, peanut seed development might be regulated by the collaboration of Ca^2+^ signal transduction pathway and hormone regulation pathway, or Ca^2+^ regulates pod development through modulating hormone since Ca^2+^ signal transduction pathway can modulate plant hormone (Yang and Komatsu, 2000; Ullanat and Jayabaskaran, 2002; Mori et al., 2006), and it also might be related to the utilization calcium of auxin and ABA pathways as both a protein binding secondary messenger and in membrane transport mechanisms that modify turgor and solute accumulation to drive cell expansion and ripening (Hocking et al., 2016).

## Author Contributions

SW and XL planned and designed the research. YL, JM, FG, SY, JZ, YG, and LC performed experiments. YL collected data and conducted analysis. YL and XL wrote the manuscript.

## Conflict of Interest Statement

The authors declare that the research was conducted in the absence of any commercial or financial relationships that could be construed as a potential conflict of interest.
